# A qualitative approach to explore the cognitive processes used by members of an adult learn-to-cycle program

**DOI:** 10.3389/fspor.2024.1220795

**Published:** 2024-08-01

**Authors:** Caitlyn Franks, Arianne C. Reis, Nicole Peel, Kylie Ann Steel

**Affiliations:** ^1^ School of Psychology, Western Sydney University, Penrith, NSW, Australia; ^2^ School of Health Science, Western Sydney University, Penrith, NSW, Australia; ^3^ THRI, Western Sydney University, Penrith, NSW, Australia; ^4^ MARCS Institute, Western Sydney University, Penrith, NSW, Australia

**Keywords:** confidence, consciousness, cycling, movement, proficiency

## Abstract

**Introduction:**

Attaining movement proficiency under various constraints is well-researched; of particular interest here is how conscious processing and self-consciousness influence learning and performance. Current research relevant to these variables e.g., reinvestment, tends to utilize quantitative methods and thus overlooks a potentially rich source of understanding. Therefore, the purpose of this study was to apply a qualitative approach to explore the cognitive processes and self-consciousness within a less practiced population of adults participating in a non-competitive leisure activity.

**Methods:**

To achieve this, eleven semi-structured interviews were conducted with adult women learning or relearning to ride a bicycle.

**Results:**

Using thematic analysis, two distinct themes were evident and corroborated those illustrated in scales such as the movement-specific reinvestment scale. These themes included Conscious Processing, Recalling Experiences and Movement Analysis, in addition to Style of Movement, and Self-Consciousness. Anxiety also emerged as an additional and relevant theme to learning a new complex movement skill.

**Discussion:**

The findings indicated that participating women attuned to their movements to the extent that there was a conscious attempt to control their movements, but less so regarding movements being altered by self-awareness. Whilst further research is required, qualitative methods provide a promising basis for exploring the cognitive process involved with the conscious process involved in learning.

## Introduction

Reinvestment is a personality trait that results in the propensity to take conscious control of pre-established knowledge, associated with performing movement skills and is instigated by increased levels of anxiety (1, 2). The movement-specific reinvestment scale (MSRS) was developed to measure reinvestment and comprises ten items, each utilizing a 6-point Likert scale (2). The two dimensions of reinvestment are Conscious motor processing (CMP), and Movement self-consciousness (MS-C) when movement is performed relative to specific high-pressure situations (3). Conscious motor processing is characterized by the deliberate control of movement or de-automatization Deikmann (4). Movement self-consciousness (MS-C), however, is the individual's awareness of how their body alters the movement style (2). The conscious awareness of movement is of particular interest when exploring reinvestment, as consciously controlling movement may impair the learning and development proficiency of high reinvesters. This tendency to reinvest is most notable when an external pressure such as stress or an incentive is present; for example, the type of pressure experienced in a competitive sports context (5, 6).

Conscious awareness of movement is common for less practiced individuals (i.e., novices) (7), even when one's movements do not require explicit attention, yet a sudden shift in conscious awareness can trigger deliberate control of movements to maintain proficient performance (8, 9). This inclination for conscious movement control, however, becomes less salient as people move from less practiced to highly practiced, as less practiced individuals tend to utilize more explicit knowledge, which is easily articulated compared to implicit knowledge (7, 10).

Deikmann (4) defined reinvestment, as the de-automatization of a previously learned skill as a result of increased attention toward the conscious control of the movement. Masters (11) extended this by stating that when individuals become aware of their movements, they divert their attention to the explicit or declarative knowledge about the task to purposefully control the execution of the task-specific movements. Consequently, this reinvestment of attention toward the conscious control of the action and perception components of movement results in decreased performance, compared to less conscious control applied to movement execution. Raab et al. (12) also state that, from an information processing perspective, memory informs decision-making whilst directing attention outward; however, when a miscommunication occurs between memory, attention, and decision-making, reinvestment is more likely. For example, when a cognitive component (e.g., attention, memory) or process (e.g., decision-making) is manipulated (e.g., a sudden inward shift in attention), the functionality of the other two is disrupted, consequently leading to a greater conscious effort to perform the skill or task (1). This is highlighted by Kinrade et al. (13), who demonstrated that when completing cognitive tasks ranging from simple to complex, followed by motor tasks where conditions also varied in complexity, those assigned to high-pressure (i.e., more complex) environments performed poorly. Moreover, Zhu et al. (14), when examining cortical activity while learning a simple finger-tapping task, found that as individuals progress from the initial stages of learning, cortical activity decreases. This indicates that conscious cognitive process becomes less necessary, and less attention demanding.

Reinvestment is more prevalent *in situ*ations where several objectives need to be fulfilled simultaneously (11). CMP and MS-C may explain why many athletes, regardless of expertise, stumble and perform poorly in certain contexts given that reinvestment is a trait thus always present (15). Indeed, decreased movement performance has been observed when individuals completed additional tasks concurrently, i.e., decrements in speed (16), accuracy (7), and overall quality of performance (1). Furthermore, Maxwell et al. (17) state that performing a secondary task hinders implicit learning, supporting the notion that multiple tasks that require attention synchronously may prompt CMP and MS-C and induce reinvestment.

In comparative studies, Beilock et al. (18) and Gray (6) found that those less practiced demonstrate a higher propensity compared to those more practiced to reinvest, except for conditions where participants may be influenced by external factors (e.g., observers or incentives). However, in instances where expertise is required to complete dual tasks in unfamiliar conditions, decrements in performance may not be influenced by expertise (2). Capio et al. (19) found that physiotherapists and their students scored the highest and similarly on the MSRS when compared to other health professionals. This suggests that regardless of expertise, CMP and MS-C scores can be similar regardless of expertise based on task difficulty and the context for the task performed. However, it was noted that scores might have been influenced by the participants' profession where attention to bodily movements is necessary, thus these findings should be interpreted with caution.

A body of research has examined the factors that contribute to the effective learning and development of movement proficiency (3, 20, 21). Historically, this has been examined through classic learning theories such as Fitts and Posner's (22) Three Stage Model, which states that successful motor learning starts at a verbal or cognitive stage before progressing to the associative and finally autonomous stage. Further, Adams (23) proposed the Closed-Loop theory, which asserts the importance of sensory feedback in processing and performing movements. In addition, Masters and Maxwell (2) state that when learning a new skill explicit and declarative knowledge is processed into long-term memory through repetition. This elicits a pre-established level of anxiety in attempting to perform without error, which leads to an automatic skill-focused mindset resulting in deliberate control of movements (3).

Studies that examine motor skill learning and reinvestment are often characteristically quantitative, though some studies have included quantifiable verbal protocols to broader experimental designs. For example, Poolton et al. (24) and Maxwell et al. (25) compared the performance of low and high reinvesters when performing a golf putting task. After the testing period participants were asked to provide verbal responses related to techniques or rules to improve performance. High reinvesters verbalised a larger number of rules and demonstrated a greater propensity to perform poorly when filmed during the transfer phase, compared to low reinvesters. Further, Maxwell et al. (17) and Zhu et al. (14) incorporated verbal protocols when investigating implicit learning and the cognitive stage of learning. However, research in this field has yet to employ a less structured qualitative approach, such as the use of semi-structured interviews. In applying a more subjective and nuanced form of inquiry it is feasible that unexpected, yet informative responses will provide further insights into the cognitive processes when less practiced or learning a new skill, and the mechanisms that may influence its efficacy. In light of this gap in the literature related to reinvestment, the current study aims to use a qualitative approach to examine the cognitive processes associated with cycling for less practiced individuals, with a specific emphasis on factors such as those associated with a propensity to reinvest. The findings from this study are expected to contribute to the qualitative cognitive-kinematic literature base and explore if the dimensions of reinvestment influence the learning or re-learning of a complex motor skill.

## Method

### Participants and ethics

Participants in this qualitative study were recruited from a metropolitan population using purposive sampling from a local cycling group that facilitates adult learn-to-cycle programs for females, followed by snowball sampling via email and word of mouth (*N* = 11, M_age_ = 43.2, SD = 13.97). To be eligible for this study, the participant needed to be over 18 years of age, and who was currently or had recently learned (or “re-learned”) to ride a bicycle. All participants who met the inclusion criteria were contacted individually by a researcher and were sent an information sheet with a consent form that outlined the requirements of the study as approved by the authors' institutional Human Research Ethics Committee [Approval Number: H14398].

### Design

Semi-structured interviews allowed the researcher to explore the topic in greater depth, with all questions presented in an open-ended format, promoting deeper discussion. The interview questions gathered data from areas including the participants’ favourite leisure activities, motivation, physical activity and leisure, embodied experiences, outlook on cycling, and reinvestment. Four questions specifically related to reinvestment were integrated into the interview and adapted from the Movement Specific Reinvestment Scale (26) to elicit qualitative responses. For instance, the MSRS movement self-consciousness items were captured from broader questions such as “*Are you concerned with how you look when you ride your bike? Has your experience of movement changed since starting to cycle? And how do you view your body and what it can do?*” Conscious motor processing-related data were extracted from responses to e.g., “*Talk me through a typical cycling session for you. What do you notice? What do you pay attention to? How are you feeling? How has your experience of cycling changed since you first learned to cycle? how does that make you feel physically? What are some of the body sensations?*” It is important to note here that as this was a qualitative approach these questions were used as a guide only and, when required, further probing questions were presented to participants to gather deeper knowledge on the topic.

### Procedure

Interviews were conducted and recorded via the online platform Zoom^©^. During interviews, participants were asked questions related to their experiences on learning or “re-learning” to cycle as adults and were encouraged to talk freely. Interviews lasted between 25 min and 3 h in duration, with one participant requesting that her interview be split between 2 days. Upon completion, interviews were transcribed verbatim either manually or with the use of a transcription tool such as Word Dictation or Otter.ai© (2016). The recordings were reviewed for accuracy, and all participants were allocated a pseudonym to maintain anonymity during analysis.

### Data analysis

The analysis of the transcripts was grounded on the principles of Braun and Clarke's (27) method of thematic analysis. NVivo^©^ 12 was utilized to assist with data analysis. Data were analyzed in a deductive manner, meaning that what constitutes a theme is determined by theory and prior research (28). Additionally, the analysis sought to identify latent themes rather than semantic themes, whereby the deductive thematic analysis examined the immersed ideas and assumptions that shaped what was being presented at face value, i.e., the semantic content (27). This form of thematic analysis was preferable given its capacity to investigate the relationship between the subjective experiences of participants and the gaps in reinvestment literature.

Braun and Clarke's (27) method of thematic analysis involve six steps. The first step includes data familiarisation, followed by the generation of initial codes, which in this case included codes such as “*Fear associated with falling*” and “*Sudden shift in attention*”. In the third step, codes that were found throughout the transcripts that showed commonality were collated into potential themes. This step resulted in 11 themes that were reviewed and narrowed down in the fourth step, resulting in two themes with two and three subthemes, respectively. The fifth step involved a final review and naming of the themes and subthemes, followed by the last step of writing up the results and their scientific relevance.

### Investigator triangulation and reflexivity

Investigator triangulation was implemented to ensure the methods of data collection and analysis were valid and free of biases (29). As such, the analysis for the current study was conducted by one researcher and reviewed and triangulated by a second member of the research team. In addition, given the qualitative nature of the study, the research team regularly reflected upon their position and how their role in the study, and their own philosophies and personal agendas may affect the research process (30, 31). This reflexive practice was particularly important during the analysis process and, together with triangulation, ensured the trustworthiness of the findings presented here.

## Results and discussion

**Figure 1:**
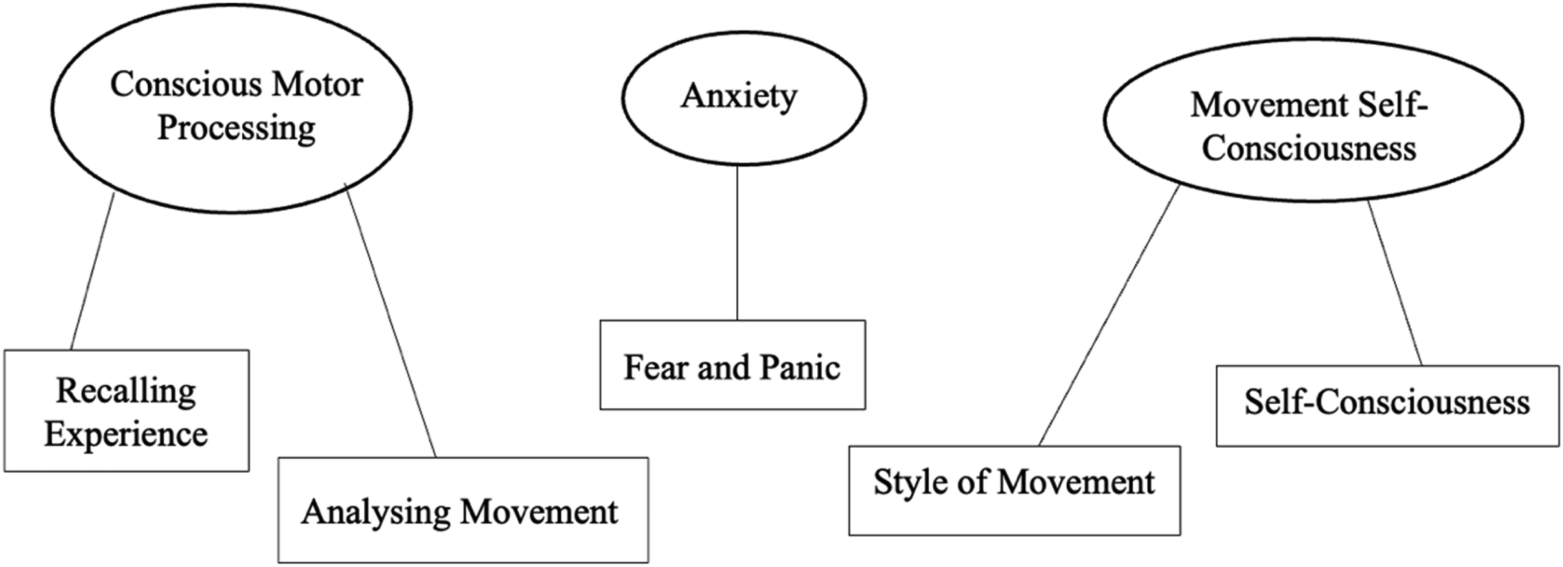
Thematic map in accordance with Braun and Clarke's thematic map (27) outlining the themes and sub-themes identified during our research.

The current study sought to explore the experiences of adults (Table 1), and in this case, females, learning how to cycle and whether factors associated with movement reinvestment impacted the learning of this complex skill. A thematic analysis (27) of the participant responses resulted in the identification of themes that align with the dimensions and triggers for reinvestment i.e., conscious motor processing, movement self-consciousness, and anxiety and are visually represented in Figure 1.

**Table 1:** Participant pseudonyms and level of experience.

Pseudonym	Age (years)	Experience (years)	Childhood experience learning to cycle
Iris	57	4	Poor
Daphne	34	1	Poor
Flora	31	3	Poor
Daisy	59	6	Good
Petunia	26	4	None
Acacia	44	2	None
Magnolia	44	7	None
Lily	62	3	Good
Poppy	30	1	Poor
Rose	26	1	Poor
Dahlia	62	6	Poor

There is a variety of ages of participants ranging from 26 to 62 years, riding experiences at the time of interview varied from 1 to 7 years. Participants also reported their experiences with cycling as a child, which were ranked across categories of none, poor and good, with the majority of participants reporting a poor experience.

Three themes were derived from the participants stories and included; conscious motor processing, anxiety, and movement self-consciousness and is overviewed in Figure 1 below using Braun and Clarkes thematic analysis (27).

Each theme included two sub-themes or elements that were derived and identified within the study as part of the final steps of analysis and an example is outlined in Table 2 below with links to participants quotes.

**Table 2:** The themes and sub-themes identified in the thematic analysis, with a supporting quote.

Theme	Subtheme	Quote
Conscious motor processing	Recalling experience Analysing movement	“*I just suddenly remembered… it just came in my memory like a flash*” “*…thoughts around my feet being on the pedals and…*”
Movement self-consciousness	Style of movement Self-consciousness	“*…for some reason when I feel like someone's watching me, I…*” “*Is that something everyone else feels or is it just me?*”
Anxiety	Fear, and panic	“*Whenever I felt fear, I'd stop the bike purposely, like my reflex would be to put both legs down*”

### Conscious motor processing

The theme of *Conscious Motor Processing* encompasses all references within the data that relate to the conscious mechanisms that inform how one performs their movements. Whilst many mechanisms were observed in the data, they could all be grouped into either sub-theme curated as *Recalling Past Experience and Analysing Movement*.

#### Recalling past experience

This subtheme is concerned with the cognitive processes involved in conscious motor processing (CMP) that were consistently highlighted by the participants, with an emphasis on attention and memory. Many participants stated that when they were first learning to cycle, they would struggle with the attentional demands of navigating the environment while also trying to avoid injuries. For example, Rose (26) said: “*You have to be careful of yourself and the environment, and I found that very challenging.*” Iris (57), who had only learned how to cycle recently, stated:

*I'm looking for potholes [laugh] yeah, I'm looking for things not to hit because I like to look around when I'm riding but I find that I'm also not wanting to fall off, so I am still being quite cautious about everything around.*

However, this demand on attentional resources decreases as movement performance increases, with attention predominantly directed towards the environment rather than the execution of movements (32). Coker (33) and Nideffer (34) term this as focus of attention which concerns the process of selectively attending to environmental information. Attentional focus can be identified based on two intersecting dimensions, width and direction (34). Width refers to the amount of information we attend to e.g., broad vs. narrow, while direction refers to the source being external or internal to the individual. The focus of attention can therefore be influenced by the demands of the task in a specific context. Implicit learning proposes that focusing on the effect of a movement on the environment (external focus) allows the motor system to operate without interference as automation increases (10). This is illustrated by Lily (62), who claimed: “*Now because I have what I think is good confidence, I can survey the area and just make sure that everything is going great.*”

Interestingly, many of the participants experienced the early phase of learning to cycle in environments that placed a high demand on external factors, thus more demanding environments may focus attention to external factors in addition to moving between broad and narrow fields of awareness. This may allow more implicit processes to enhance skill development (10). Conversely, it is possible that the method for distributing attention was dependent on skill level and, perhaps, the anxiety that these women experienced. That is, the fear of being injured, makes the progression to an external focus difficult as the instinct for self-preservation overpowers the demand for attentional resources (35). Lily attests to this notion, claiming that the progression for her was “*Oh, couple of years. Seriously, it's been, it's not, umm, an instant thing.*”

Previous experiences with cycling also had a significant effect on re-learning to cycle as an adult, with many participants recounting memories of knowing how to ride a bike at an earlier time in their lives. Poppy (30) stated: “*I'm pretty sure like, I knew how to ride, like, the basics before I started*”*;* Dahlia (62) said: “*I knew how to ride even though I can't really remember how*”*;* while Daphne (34) recounted:

*I just clicked like when I reached the end and I was getting ready to do the turn, I just suddenly remembered that I have to shift my weight a little bit to the outside of the turn and I don't know, it just came in my memory like a flash and then I sort of did that and it worked, and I made a turn.*

This is interesting as the reinvestment theory assumes that having implicit knowledge of movements would indicate that performance would be more autonomous; however, the participants still experienced difficulties with learning cycling movements later in life. A reason that was commonly cited in the interviews were negative experiences associated with cycling in the past, for example, “*I remember my sister, like, cycling down this steep path and she fell over and I was like* ‘*I don't wanna do that*’” (Rose, 26). Studies of reinvestment have found that a history of an injury, particularly falling, positively correlates with higher scores on CMP items on the MSRS (35). Despite possessing the knowledge of how to execute movements, a history of injuring oneself while executing movements can inadvertently result in actions becoming a conscious activity (36). Additionally, this could be explained by negativity bias, whereby negative information about a stimulus is more accessible than positive or neutral information (37). Therefore, despite having declarative knowledge of how to ride a bicycle, a negative memory of cycling can overpower the knowledge and increase the tendency to reinvest.

#### Analysing movement

The subtheme analyzing movement explores how participants reflect on their movements. During the early stages of learning, participants reported that their movements were more error-prone and guided by their fear of injury. Acacia (44) spoke on this idea, saying: “*I was still wobbly, I would still do multiple false starts, and whenever I felt fear, I'd stop the bike purposely, like my reflex would be to put both legs down.*” Flora (31) also stated: “*thoughts around my feet being on the pedals. And mostly… guarding in case I needed to brake suddenly.*”

Masters (11) posits that the beginning stages of learning are slow and require more conscious awareness as the knowledge required for performance is explicit [e.g., “*I didn't have the proper knowledge to do it.*” Lily (62)]. Neuroscience research provides an additional neurobiological explanation for this assertion. Denneman et al. (5), and Zhu et al. (38), measured various components of brain activity related to learning and performing movement skills and found that as movement automaticity increases, the demands on relevant interconnected regions and functionality within the brain, decreases. As such greater cognitive effort can be applied to factors such as the environment, or if within a sport context, tactical demands (39).

Selected learning theories also suggest that if learning and skills are acquired at a slower pace, this could create a consistently proficient long-term performance (40, 41). For instance, the Desirable Difficulties Framework (42) and the Challenge Point Framework (43) both assert that if the difficulty of the skill matches the level of prior knowledge and expertise of the learner then learning will be successful. As some participants had a history of cycling in their childhood, these frameworks provide a plausible explanation as to why they continued to cycle despite their fear. That is the level of difficulty they experienced when they were re-learning matched what they already knew. Even Daisy (59), who had some knowledge and experience of cycling before joining the group, stated: “*I did learn some tips about the best way to turn and, you know, issue standing and pedaling how that affects your body, and [laughs] it takes a lot out of you*”*.*

Over half of the participants in this study reported that they had been cycling for a minimum of 1 year and would be classed as community or leisurely cyclists with no regular pattern of hours committed to cycling as a sport. Furthermore, they stated that cycling had become relatively autonomous, proficient, and now requires little conscious effort. For instance, Poppy (30) stated: “*when I just get back on the bike, it's just like, never really forget how to ride a bike*”*,* and Iris (57) commented:

*I felt by the end of it that we were riding along and skidding out and doing this really cool burnouts that I just did not think I would get to the point of even wanting to try that, but it was very cool fun.*

The responses from participants who had been cycling longer corroborate findings in learning literature, in that, as learning progresses the information required for movements associated with cycling becomes implicit and, thus, individuals can perform without needing to retrieve explicit information about the movements (2, 17). For instance, Acacia (44) claimed: “*And you know, that trial and error, you know, and I just kept at it and kept at it until eventually, um you know, I started riding.*” In many forms of skill learning, including athletic skills, Goddard (44) posits that to learn a skill successfully the learner requires effective demonstration, practice, and feedback over an extended period. Most of the women in the study had learned to cycle through a program designed to incorporate these strategies. Even Daphne (34), who was self-taught, had the assistance of friends:

*He was telling me how I can see from the position of the chain what gear it is and even when it's stationed, you need to be pedalling for the gears to move and change so the technical side of it he was telling me.*

The data gathered from these interviews provide support for the established literature describing the cognitive processes involved in learning, in addition to the impact that conscious control of movement can have on learning where there may be a propensity to reinvest.

##### Movement self-consciousness

The theme of *Movement Self-Consciousness* encapsulates any references in the data to the participant being aware of their bodies. This theme also examines if the awareness these women experienced may have shaped their performance in any way, with two subthemes surfacing during the analysis: *Style of Movement,* and *Self-Consciousness.*

#### Style of movement

This subtheme refers to how participants felt about the way they appeared when moving in addition to instances of confidence. Many of the participants in the study reported that cycling confidence evolved in tandem with their movement proficiency. For example, Poppy (30) commented: “*I would get nervous when there's, like, so many people around, but over time, you just build confidence*”*;* and Dahlia (62) commented on how her changes in movement ability enabled participation in more challenging cycling contexts:

*I said […]? I'm never gonna ride on the road, just never expect me to go on a ride. I'm not gonna do it, it's too scary and she reminds me that I said that every time we go on the road now.*

Participant responses showed that concerns about their bodies and how they moved diminished as confidence and proficiency developed. It is also possible that as we age, we focus more on movement control rather than on movement style (35, 45, 46). For example, Malhotra et al. (3) demonstrated that the demand to be aware of the surrounding environment leaves fewer attentional resources for other factors (e.g., concern with movement style). Therefore, participants in the current study may not have had the attentional capacity to be concerned with the style of movement while becoming comfortable with navigating the environment. This process, however, took time as the anxiety associated with injury ensured an internal focus on their movements e.g., “*I'm probably feeling it now [confidence] but that's three and half years, so maybe three years I think it's been it hasn't come easily*” Iris (57). It is, therefore, feasible that the confidence grows in tandem with movement proficiency and could be a protective factor against movement self-consciousness.

When recounting their experiences of cycling, several women referred to becoming attuned to their movements as their performance increased: “*Is that something everyone else feels or is it just me?*” Rose (26). Daisy (59) talks about this notion, saying:

*I am quite aware of my body when I'm cycling. If I'm going up a hill, I can tell you which parts of my body working the hardest, so it'll be like my glutes, I can really feel they're engaged. I can feel that I am probably breathing harder if it's cold, because I quite often ride it really early in the morning and I'm heading up the hill.*

In fact, some women stated that they enjoyed being aware of their movements, with Flora (31) commenting “*I like the pedaling and that repetitive and the feeling of your legs actually working.*”*,* and Dahlia (62) saying “*I love the motion, I love the movement, I love the floating/flying sensation. I love the pedaling. I know that probably sounds weird, but I love the rhythm.*”

This is relevant to the reinvestment theory, as the responses imply that although the participants had an awareness of their movements whilst cycling, they did not indicate that this had an impact on their performance, which contradicts assertions of reinvestment and the impact of internal focus of attention. One explanation is put forward by Toner and Moran (47) and Toner et al. (48), who suggest that skilled individuals can utilize bodily awareness to facilitate improvement during motor performance, sometimes referred to as reflective awareness. Alternatively, embodiment research would argue that knowledge comes from the physical senses and that these women are learning through the interaction between the body and the bicycle (49). While some participant responses do not corroborate some aspects of reinvestment theory, they can be explained based on alternative theories of kinematics or experiences of embodiment thus suggesting continued investigation is required.

#### Self-consciousness

This subtheme concerned perceptions of the body related to movement and body image. Analysis of the responses showed that participants who experienced concerns about their body image did so during the initial stages of learning. Daphne (34), who is new to cycling, stated: “*The first time I was about to ride like* ‘*oh my God I look so lame*’*,* and Dahlia (62) commented: ‘*I've always had that image of a clown on one of those little, tiny bicycles in the circus and I didn't like that. I didn't want to look like that.*’” These concerns about body movement and image, similar to anxiety, dissipated as movement proficiency increased: “*The feel of awkwardness is not bad enough to stop trying*” Daphne (34).

Self-consciousness about their body image and movement did not appear to hinder or impact performance. This may be attributed to an adult demographic who are less impacted by movement self-consciousness compared to conscious motor processing (46). This finding is also consistent with the consensus that self-consciousness does not increase the processing of movement information as less practiced individuals are already utilizing explicit knowledge (11, 50). The anxiety to perform without error when learning a new skill may also leave little attentional capacity for body image concerns (35). Indifference to body image could also be an attribute of personality, e.g., “*I'm not one to worry too much about that ever [laugh] in any situation for that matter*” Magnolia (44), or the possibility that those with body image concerns did not engage in this activity in the first instance. Regardless, it can be suggested that these women showed little or no concern for their body image, which in turn had minimal impact on motor performance.

##### Anxiety

Anxiety is the physiological and psychological response to stimuli or environments that induce emotional distress and can either be elicited by perceived threats (state anxiety) or have its origins in personality or genetic predispositions (trait anxiety) (51). In the context of this research, anxiety was examined in relation to its impact on inducing movement reinvestment when learning to cycle. Ten of the eleven participants commented that they experienced anxiety while learning how to cycle, especially when they first started learning. Several participants expressed how it felt when first learning to cycle: Acacia (44) said “*I was petrified to get on a bike*”*,* and Magnolia (44) had a similar response, “I *was afraid to start*” and “*At the start, it was a bit daunting*”*.*

Many participants also commented that the environment in which they learned to cycle would often elicit feelings of anxiety. Flora (31) commented “*…the part where I had the most fear was going over this really thin bridge with lots of pedestrians.*” Moreover, Poppy (30) stated: “*the first day was a bit nerve-wracking because I had to ride on, like, shared paths and shared paths that… there might be people walking*”*,* and Rose (26): “*Yeah, I don't know if [I] would cycle if I had a lot of people cycling with me. I don't know if I would. I don't think I'm that confident.*” The learning environment elicited base levels of anxiety before starting the task, coupled with the demand for psychomotor proficiency and spatial awareness in new and constrained contexts. Nieuwenhuys-Oudejans’ Integrated Model of Anxiety and Perceptual Motor Performance (52) further explains this in terms of how stimuli that are perceived as threatening determine anxiety and the effectiveness of one's movements. Young et al. (46) noted that a common form of anxiety-induced performance decrement was associated with crowd pressure. Thus, it can be said that environmental factors that threatened movement proficiency were a viable contribution to base levels of anxiety when the participants were first learning how to cycle.

The fear of hurting oneself also appeared to elicit anxiety for these women, with nine admitting that they feared sustaining an injury while cycling. Daphne (34), in response to how she initially felt when she jumped on her bike, commented: “*Then, when I actually come to do it, it's just like, I'm just like, I'm gonna fall. Like, right away even before I'm on the bike.*” Similarly, Acacia (44) stated: “*I knew in my head I'm afraid to get on a bike because I didn't wanna fall*”*.* This fear may be age-related, in the sense that some participants alluded to being afraid that an injury may be more detrimental to meeting the demands and commitments of their everyday lives compared to when they were younger. Acacia (44), for instance, said: “*I guess as an adult, you know, in my 40s, I'd get on a bike, and I'd have this fear* ‘*I'm gonna fall, I'm gonna fall, I'm gonna break my arm or I'm gonna break my leg*’”*.* Magnolia (44) also commented: “*as an adult, you have a lot more fear in you than you do as a child.*” Even Petunia (26), one of the youngest participants, hints at this, stating:

*…learning in your 20s it's pretty good cause you're probably pretty physically fit and you still know how to do everything and, in your muscles, and joints aren't really sore, and accidents or injuries or anything like that.*

Fear of injury is particularly common in older adults as motor performance begins to regress (53). This regression means that to maintain balance more attentional resources are required, whilst also being aware of anything that threatens to disrupt one's balance (46). As cycling requires more balance than standing or walking, it makes the possibility of injuries higher. Further, given injuries resulting from falls are a leading cause of bone fractures, especially as we age (46), any concerns exhibited by participants in the current study are justifiable.

##### Limitations and implications

The current study provided further evidence that demonstrates the impact of dimensions of reinvestment on the execution of movement skills, with an emphasis on individuals who are less practiced with a complex movement skill. Future research could explore factors such as falls history and its perceived effect on learning. Further, it may have been beneficial to administer the MSRS before the interviews as the responses could be utilized to find congruencies and discrepancies within the results. Finally, conducting the interviews over Zoom was a considerable limitation in the sense that the intent of the information shared may have been missed due to technical issues, and rapport could not be established in the same way that it would have been if interviews were conducted face-to-face.

Given that explorative, qualitative research in the field of reinvestment is scarce, the approach used in this study should be replicated in future studies and comparing the findings to quantitative methods, thus using a mixed methods approach. In addition, future studies could include a larger sample with greater variance in skill ability, while using a longitudinal approach, to extend our understanding of the factors that influence reinvestment (7, 54, 55).

## Conclusions

This study aimed to examine whether the characteristics associated with the dimensions of reinvestment, impacted the development of movement proficiency when learning or re-learning to cycle. Using a sequential thematic analysis based on semi-structured interviews, it was clear that participants in this study displayed reinvestment tendencies with respect to taking deliberate control of movement execution (CMP), but not with respect to being concerned about their bodies or style of movement (MS-C). Further, anxiety related to the environment and being injured, and the cognitive processes such as attention and memory, contributed to CMP and consequently learning. Regarding MS-C, it was found that confidence and body image concerns were positively altered as performance improved, and whilst the women were attuned to their movements this did not impact their performance. This finding does not corroborate the commonly reported findings in reinvestment literature and requires further exploration. This study applied an underutilized method to extend our understanding of the underlying process involved in reinvestment with a population previously unexplored and warrants further exploration. In conclusion, this study can serve as an impetus for further research into reinvestment in the correlation between psychology and kinematics, while using qualitative and mixed methods.

## Data Availability

The datasets presented in this article are not readily available because these were interviews they will not be made available outside of the listed research group to maintain participant privacy. Requests to access the datasets should be directed to k.steel@westernsydney.edu.au.
